# Sex in Industry

**Published:** 1875-08

**Authors:** 


*Sex in Industry; a Plea for the Working Girl.* By Azel Ames, Jr., M. D. Boston: J. R. Osgood & Co., 1875; pp. 158.

The interest awakened in the behalf of the female population and their relations to the various modes of education, by Dr. Clarke, has developed itself in various ways. Taking Dr. Clarke's "Sex in Education as his model, Dr. Ames has endeavored to make a similar plea for the female sex compelled to follow the various industrial pursuits.

In education the question propounded by Dr. Clarke, is not *shall* woman learn the alphabet, but *how* shall she learn it, and in view of the same physiological facts Dr. Ames asks, what shall woman do and what not do, and how shall she least harmfully do that which she may undertake. More than ten per cent. of the working women of the United States are under fifteen years of age. These in the aggregate are something over 191,000 as shown by the United States Census of 1870. Dr. Ames says that the condition of these females is far worse than that of the student, for the reason that the method of study has only to be changed to produce the desired reform, while with the operative in any branch it often happens that the character of the work as well as the method is wrong.

Of the large number of girl-workers under fifteen, it is highly probable that a large per cent. have not established their menstrual periods. Dr Clarke in speaking of this subject says:—A careless management of this function, at any period of life during its existence, is apt to be followed by consequences that may be serious; but a neglect of it during the epoch of development, that is, from the age of fourteen to eighteen or twenty, not only produces great evil at the time of neglect, but leaves a large legacy of evil to the future." In pointing out the evil effects of constrained position, muscular effort and brain work, the same author says that the female operative suffers less from these influences than the student for the reasons:—1st. The female operative as a rule has passed through the critical epoch of a woman's life. 2d. She has less brain work than the student.

In answer to the first of these statements, Dr. Ames quotes the statistics given above. It is also obvious that in the intricacy of the modern machinery employed and the many mental processes demanded in certain employments, demands a large share of brain work, less, no doubt in the aggregate than put forth by the student, but still of considerable importance. "Again," Dr. Ames says:—"There are conditions connected with the acts of cerebration in the operative that in and of themselves are potent for evil; as, the monotony, depression, bodily fatigue, and "constrained position," few of which find their counterparts in ordinary student toil."

Dr. Beard says; Brain-workers live under better sanitary conditions than muscle-workers. Brain-workers can adapt their labor to their moods and hours, and periods of greatest capacity better than muscle-workers." Death rate tables of the three classes, gentry, tradesmen and operatives, give surprising results against the operatives both in longevity and youthful deaths.

The loss to the State by the sickness or death of the operative is a direct loss of income. "If by indiscretions of educational methods the young female sacrifices life or health, the loss, though great, is but that, so far as the state is concerned, of an unproductive unit." With the working girl the

Commonwealth loses her labor, and herself as a unit in the maintenance of society. The student may have given promise of a life of usefulness, the worker has already begun it.

The author gives a tabular view of the loss, first to the individual, and then the state, produced by any form of occupation which hinders female health and developement. The errors of employment that produce these results are first, the early age at which girls are put to work, second, working them too many hours at regular and prolonged tasks, third, their employment at tasks which cannot be undertaken without injury, except by fully developed women; fourth, allowing no rest when their physical conditions demand it, i. e. during the menstrual period.

The employment of girls and young women is shown to be of too great duration, and requiring too close attention, and frequently attended by bad hygienic surroundings.

The writer glances at the more important and ordinary occupations in which females are employed, and gives numerous illustrations of the bad results arising from the employment of young females. He says that there are but two ways in which evils of this nature are to be met. "They are the diffusion of sound intelligence bearing thereon, and the enactment and enforcement of efficient repressing laws."

A few suggestions as to further enactments in this regard, and remarks upon the position of the working woman, close a volume, which, though small, is full of suggestive material, presented in an admirable manner.

